# Computational identification of RNA functional determinants by three-dimensional quantitative structure–activity relationships

**DOI:** 10.1093/nar/gku816

**Published:** 2014-09-08

**Authors:** Marc-Frédérick Blanchet, Karine St-Onge, Véronique Lisi, Julie Robitaille, Sylvie Hamel, François Major

**Affiliations:** 1 Institute for Research in Immunology and Cancer, Université de Montréal, PO Box 6128, Downtown Station, Montréal, Québec H3C 3J7, Canada; 2 Department of Computer Science and Operations Research, Université de Montréal, PO Box 6128, Downtown Station, Montréal, Québec H3C 3J7, Canada

## Abstract

Anti-infection drugs target vital functions of infectious agents, including their ribosome and other essential non-coding RNAs. One of the reasons infectious agents become resistant to drugs is due to mutations that eliminate drug-binding affinity while maintaining vital elements. Identifying these elements is based on the determination of viable and lethal mutants and associated structures. However, determining the structure of enough mutants at high resolution is not always possible. Here, we introduce a new computational method, MC-3DQSAR, to determine the vital elements of target RNA structure from mutagenesis and available high-resolution data. We applied the method to further characterize the structural determinants of the bacterial 23S ribosomal RNA sarcin–ricin loop (SRL), as well as those of the lead-activated and hammerhead ribozymes. The method was accurate in confirming experimentally determined essential structural elements and predicting the viability of new SRL variants, which were either observed in bacteria or validated in bacterial growth assays. Our results indicate that MC-3DQSAR could be used systematically to evaluate the drug-target potentials of any RNA sites using current high-resolution structural data.

## INTRODUCTION

Across the world, people are dying from bacterial infections, which formerly we were able to manage with antibiotics. Constant genetic variations in bacterial strains result in antibiotic resistance and in a constant need for new antibiotics (1). The structures of several prokaryotic and eukaryotic ribosomes free and bound to antibiotics have been determined at high resolution (2,3). These structures revealed important molecular details about how some antibiotics bind and affect ribosome function, how mutations can lead to antibiotic resistance and offer new insights into designing new antibiotics (4,5). Ribosome-targeting antibiotics have dominated the market because the ribosome is the main component of protein biosynthesis, and bacterial ribosomes contain RNA structural motifs that are absent in humans.

Despite access to high-resolution structures, identifying structural motifs and functional determinants of bacterial growth requires additional data. For instance, mutagenesis experiments produce invaluable information by providing positive, but also negative, examples, which combined with high-resolution structures have been shown crucial to identify nucleotides interactions and chemical groups involved in vital functions of bacterial ribosomal RNA (rRNA). The comparison of crystal structures of viable and lethal sequence variants in *Escherichia coli* provided further insights into structural elements of the 23S rRNA involved in the recognition and binding to the elongation factor G (EF-G) (6–8).

Obtaining sequence variation and bacterial growth data is inexpensive compared to resolving high-resolution structures. Given the recent improvements in RNA tertiary (3D) structure prediction methods (9–14), we evaluated if they could be integrated to quantitative structure–activity relationship (QSAR) approaches to automate the identification of ribosomal determinants of bacterial growth. QSAR methods are usually employed to optimize the chemistry of ligands bound to receptors in the context of rational drug design. They have been used to optimize protein primary (15) and topological structure (16,17), as well as topological descriptors of chemical structures, e.g. anticancer agents (18).

QSAR methods have been also applied to RNA. They have been used successfully to probe the anticancer activity of synthesized nucleosides (19), to predict the local binding affinity constants between a specific nucleotide and an antibiotic (20) or to recognize miRNAs involved in early states of development and stem cells (21). The predictor variables used to model these RNA QSAR approaches range from secondary (2D) structure descriptors like chemical topology (i.e. planar representation of atoms and their chemical bonds) (19) to vectors of numerical values representing nucleotide properties, such as experimental molar absorption coefficients, single excitation energies and oscillation strength values (20). Finally, RNA QSAR was applied to determine thermodynamic molecular descriptors based on free energy, entropy and melting temperature (21). All those models were calibrated using linear discriminant analysis techniques and validated using either cross-validation techniques (19), leave-one-out jack-knife experiments (20) or receiver operating characteristic curve analysis (21).

**Figure 1.**
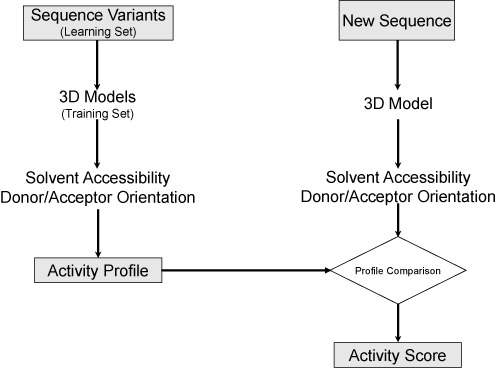
MC-3DQSAR steps. A set of 3D models is built for each sequence of the learning set by using atomic superimposition of substituted bases in a template of a reference high-resolution structure, or by 3D modeling. The models constitute the training set. The exposed area of each atomic group is computed using pymol and a probe water of radius 1.4 Å. The solvent accessible groups are considered potential determinants if they are present in all positive examples of the training set and absent in at least one negative example. Determining the activity of a new sequence variant consists in building its 3D structure, computing the solvent accessibility of its chemical groups and comparing its profile to the activity profile.

Here, we introduce an RNA 3D structure QSAR method, MC-3DQSAR, to deduce structural determinants associated with a given function, such as binding to a particular factor, participation to a catalytic reaction or bacterial growth. We start from a set of positive and negative sequence variants. For each variant, we use a representative all-atom 3D structure, either computationally predicted or experimentally determined (training set). For each heavy atom of the bases in each structure, we determine its hydrogen donor–acceptor potential and solvent accessibility. From this information, we define the activity profile of a training set, which we use in predicting the activity of new sequence variants. Given a new sequence, we compute its corresponding 3D structure and an activity score based on its similarity with the activity profile derived from the training set (see the Materials and Methods section and Figure 1).

We applied MC-3DQSAR to the bacterial 23S rRNA sarcin–ricin loop (SRL), which is located in one of the longest conserved sequences in the ribosome (22). The SRL is highly conserved across ribosomes and is a well-known region of deleterious mutations. It is recognized and bound by the α-sarcin and ricin toxins, which inactivate protein synthesis by blocking its interaction with the EF-G (8,23). Since the structure and function of the SRL has been largely studied, it makes it an excellent benchmark to study its QSAR. We also applied this approach to the lead-activated ribozyme (leadzyme) (24,25) and hammerhead ribozyme (26,27).

## MATERIALS AND METHODS

### Learning sets

#### 23S rRNA SRL

We used a total of 31 different SRL sequences (Table 1). The SRL from the 23S rRNA in *E. coli* was used as a reference. Eleven variants, IDs #01 to #11, were taken from the literature and along with the *E. coli* were used as the learning set. The viability of these sequences was tested experimentally in bacterial growth assays (7,28–30). Eight variant sequences were extracted from a 23S rRNA bacterial alignment, IDs #12 to #19. Finally, we evaluated the remaining 11 variations of the junction base pair (C2658:G2663) IDs #20 to #30. We experimentally tested the viability of six new variants: IDs #12, #13, #19, #26, #28 and #30.

**Table 1.**
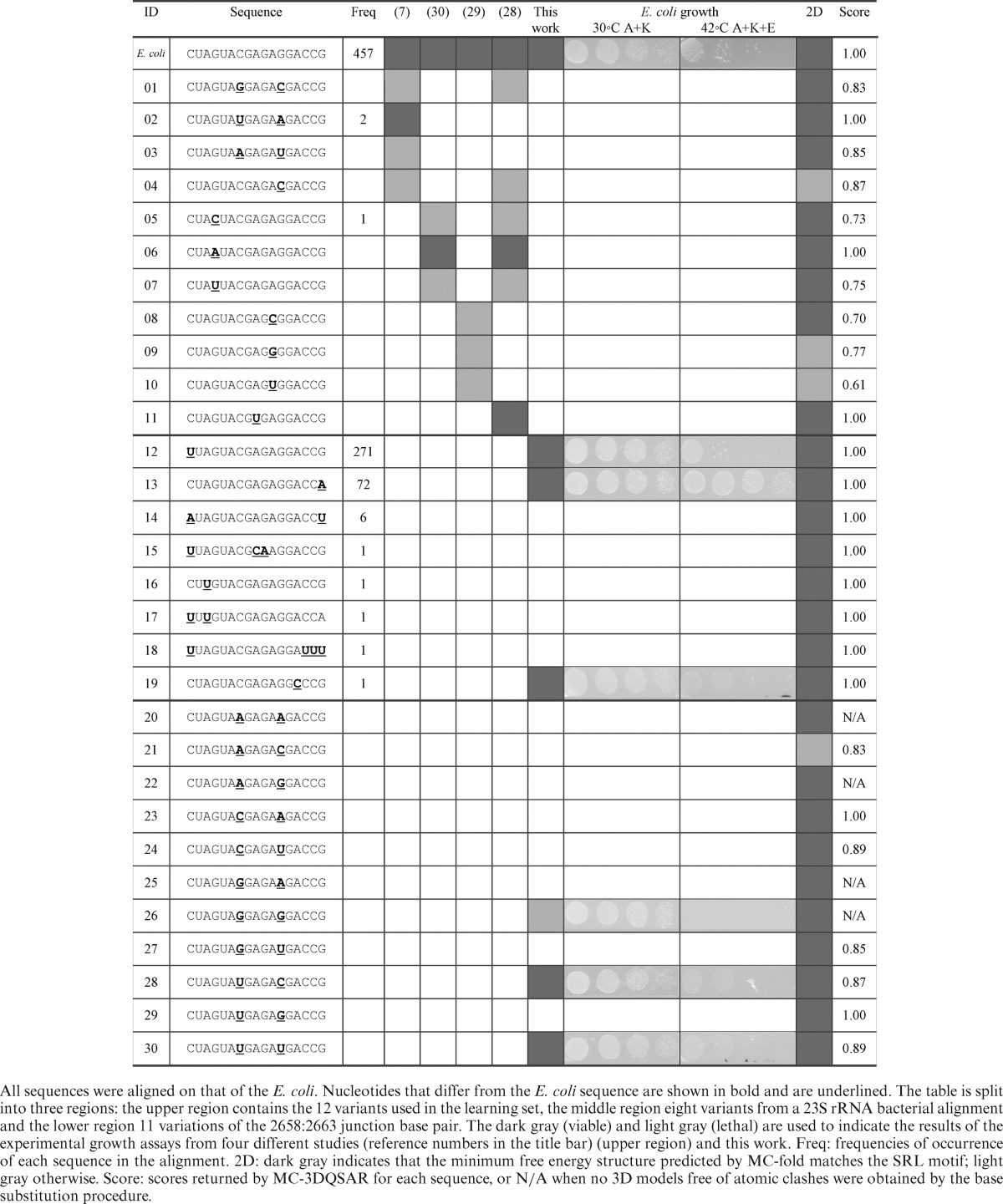
SRL variants

#### Leadzyme

We used 35 leadzyme sequences (Table 2). The reference sequence was taken from Uhlenbeck and colleagues (ID seed) (24). The learning set was constituted of 34 sequence variants: #01 to #08 considered active, and #09 to #34 considered inactive. The cleavage activity of all sequence variants was tested *in vitro* (24).

**Table 2.**
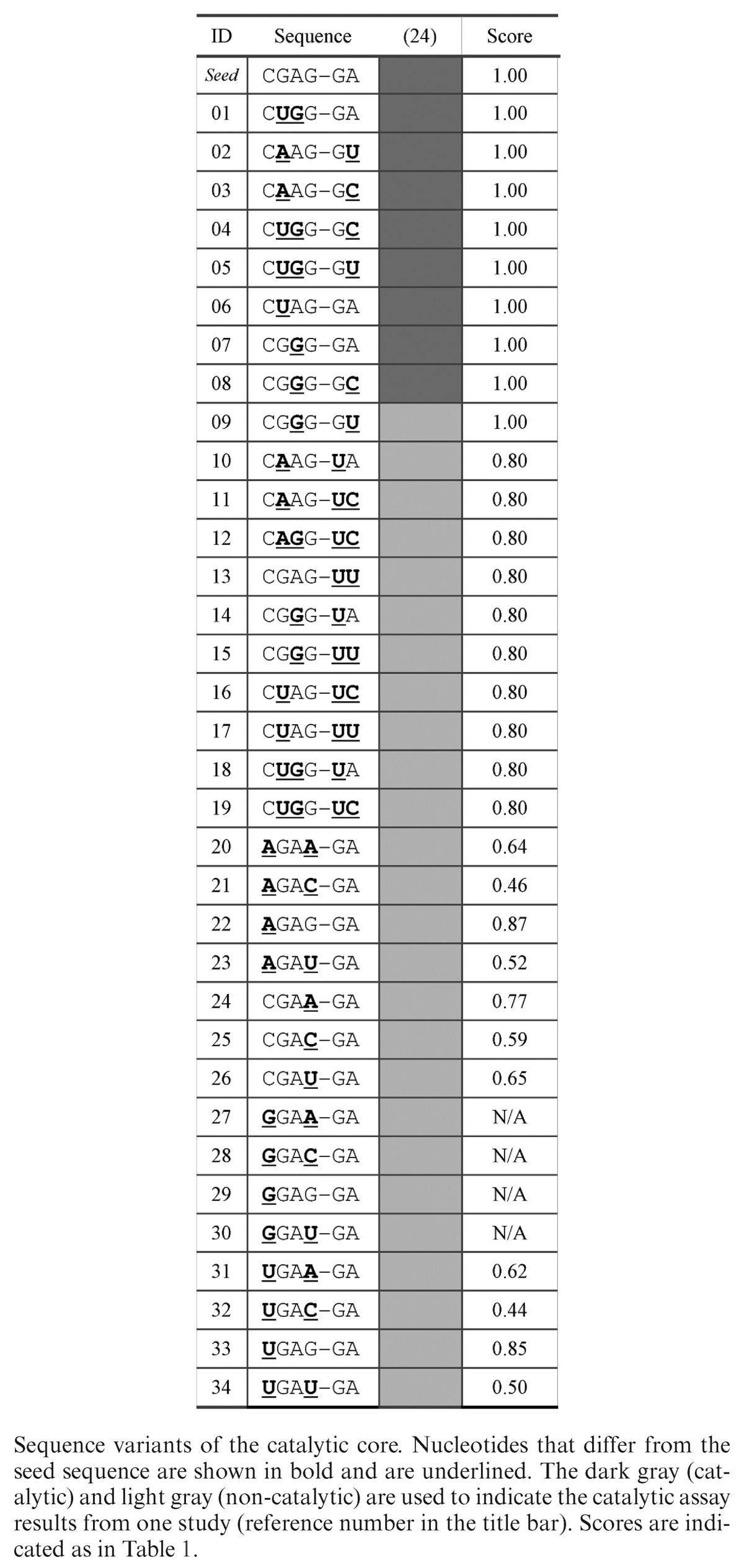
Leadzyme variants

#### Hammerhead ribozyme

We used 55 hammerhead ribozyme sequences (Table 3). The reference sequence was taken from Uhlenbeck and colleagues (26). The learning set was constituted of 12 active and 43 inactive sequence variants tested *in vitro* (26).

**Table 3.**
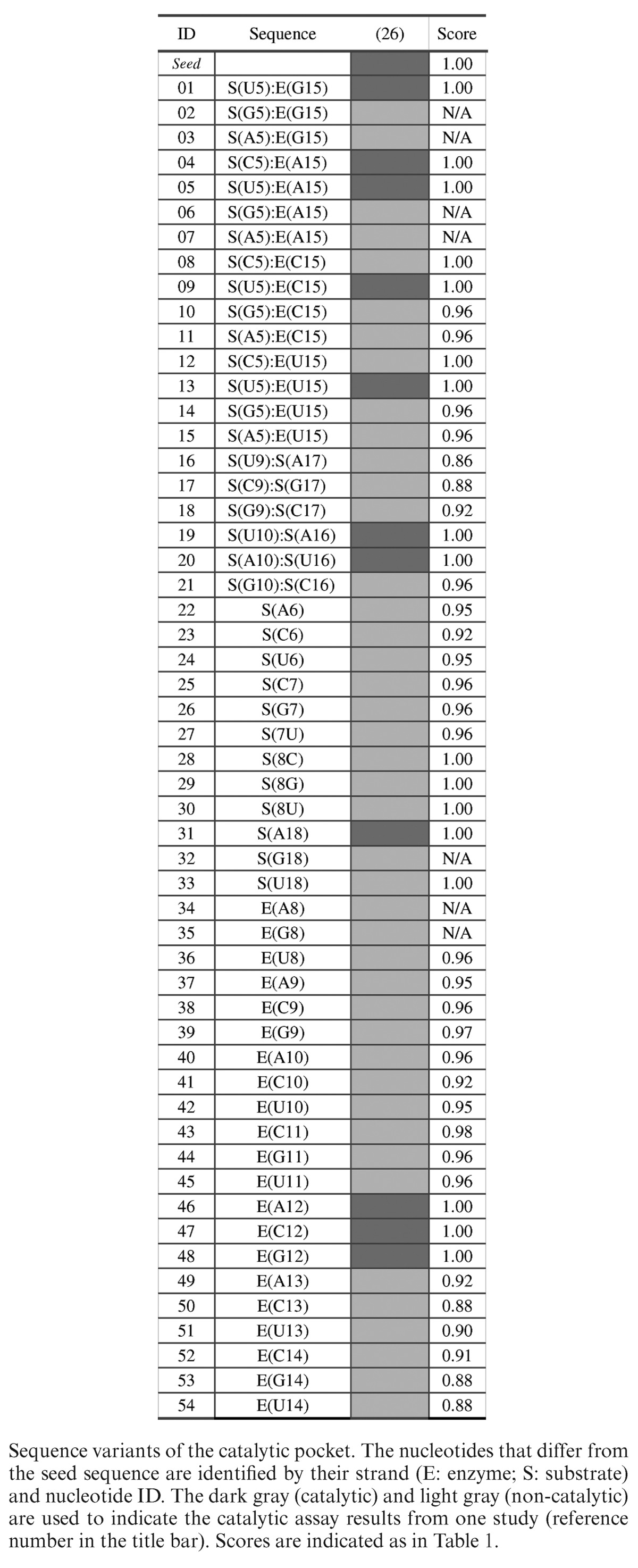
Hammerhead ribozyme variants

### Base pairing and stacking nomenclature

We used the Leontis–Westhof base pairing nomenclature (31). The canonical Watson–Crick base pairs are indicated by black circles, sugar edge by triangles and Hoogsteen edge by squares. Black-filled symbols indicate *cis* base pairs and empty symbols *trans*. The dark line indicates the presence of phosphodiester linkage. The base stacking is indicated using the Major–Thibault notation (32): >> forward/backward, <> outward and >< inward.

### Bacterial strains, plasmids and mutagenesis

The Wool lab at the University of Chicago generously provided us with the *E. coli* DH1 strain containing the pcI857 plasmid (referred to as DH1/cI) and the pLK45 plasmid. The pcI857 plasmid contains the thermolabile λcI repressor and a kanamycin resistance gene. The pLK45 plasmid contains an ampicillin selection marker and the rrnB operon under the control of the λP_L_ promoter, which together permit the expression of the 23S containing a mutation conferring erythromycin resistance (A2058G). At 30°C, the thermolabile λcI repressor inhibits the transcription of the rRNA contained in pLK45 via the promoter IP_L_. At 42°C, the repressor is inactivated, which induces the expression of the pLK45 plasmid encoded rRNA. The addition of erythromycin inhibits the natural 23S, leaving the plasmid expressed rRNA uniquely. Mutagenesis of the 23S ribosome was performed using the QuikChange II XL mutagenesis kit from Agilent Technologies according to the manufacturer's instructions.

### Growth assays

DHI/cI cells with wild type or mutant 23S rRNA were grown in LB (Luria Broth) containing 50-μg/ml ampicillin and 30-μg/ml kanamycin at 30°C to an absorbance of 0.6 at 650 nm. Five serial dilutions (10^−1^ to 10^−5^) were made and applied in 7-μl drops on agar plates containing 50-μg/ml ampicillin and 30-μg/ml kanamycin, or 50-μg/ml ampicillin, 50-μg/ml kanamycin and 50-μg/ml erythromycin. The plates were incubated at 30°C (ampicillin and kanamycin) or 42°C (ampicillin and kanamycin–ampicillin, kanamycin and erythromycin) for 16–20 h. Experiments were performed twice in technical duplicates, and the results are shown in Table 1.

### MC-3DQSAR

#### 3D structure building and selection

The SRL 3D structure of the *E. coli* 23S rRNA (PDB ID 2AWB, nucleotides B2652–B2668) was chosen as a reference structure. For each variant in the learning set, we built a 3D model that conserved the positions and orientations of the bases using atomic superimposition. This gave us a training set composed of four viable and eight lethal 3D structures. Note that this step can alternatively be achieved by using 3D structure prediction software, such as MC-Sym (13) or modeRNA (14), for instance. Similarly, a crystal structure was used for the atomic coordinates of the seed leadzyme (PDB ID 1NUJ) (25), as well as for the atomic coordinates of the seed hammerhead ribozyme (PDB ID 2OEU) (27). The 3D models of the leadzyme and hammerhead ribozyme of the learning set were built in the same manner as was done for the SRL. All 3D models are available online at major.iric.ca/MC-3DQSAR.

#### Activity profile computation

Using the 3D models of a training set, we identify the heavy atoms that are accessible to the solvent using pymol and a probe water of radius 1.4 Å (33). We add the exposed surface of the hydrogen atoms (H) attached to heavy atoms. For each structure in the training set, we keep the position, potential role of H-donor/acceptor and a unit vector defining the direction of potential participation in H-bonding formation of these atoms (total surface including attached H atoms greater than or equal to 0.25 Å^2^). These atoms in the seed are considered potential determinants that define the initial activity profile; note that the activity profile could be defined from any positive example of the training set. The activity profile is defined by the determinants present in all positive examples of the training set and absent in at least one negative example.

To identify the determinants present in all positive examples, we best align the 3D structures of the training set; note that models built by atomic superimposition over the seed structure are already best aligned. Then, for each potential determinant, we compute matching scores with the exposed atoms in all other positive variants of the training set. The scores are initialized to 1.0 if their H-bonding roles match, or 0.0 if not. We adjust the scores by dividing by the square of the inter-atomic distance if it is over 1.0 Å; the score is kept as it if the distance is below or equal to 1.0 Å. Multiplying with the scalar product between the potential H-bonding unit vectors further scales the score. If multiple H-bonding directions are possible (e.g. O6 of the guanine), we keep the best score. The determinants for which no matching score over or equal to 0.75 (threshold) is considered unrelated to the activity are removed from the viability profile. For each remaining potential determinant, if no matching heavy atom and role is found in at least one of the negative variants, then we keep it as an activity determinant.

To determine the parameters, we explored a range of values. We tested areas between 0 and 1.0 Å^2^, by increments of 0.25 Å^2^, and match thresholds of 0.05, 0.25, 0.50, 0.75 and 0.95. For match thresholds of 0.75 and above, we were able to separate the active (score of 1.0) from the inactive variants (score under 1.0) for all tested area values. We thus elected to use a threshold of 0.75 Å^2^, which allows for the loosest fit possible that perfectly separates active from inactive variants. To focus on the exposed atoms (forming the active interface), we selected the minimum tested non-zero exposed area of 0.25 Å^2^.

#### Activity prediction

For a new variant 3D structure, we compute an activity score defined by the sum of the matching scores for each determinant of the activity profile, divided by the number of determinants (resulting in a score between 0.0 and 1.0).

### Molecular graphics

The molecular images were generated using pymol (33). The electrostatics surfaces were obtained using pdb2pqr (34,35) and APBS (36), and the rendering using povray 3.6 (Persistence of Vision Pty, Ltd 2004).

## RESULTS

### SRL

**Figure 2.**
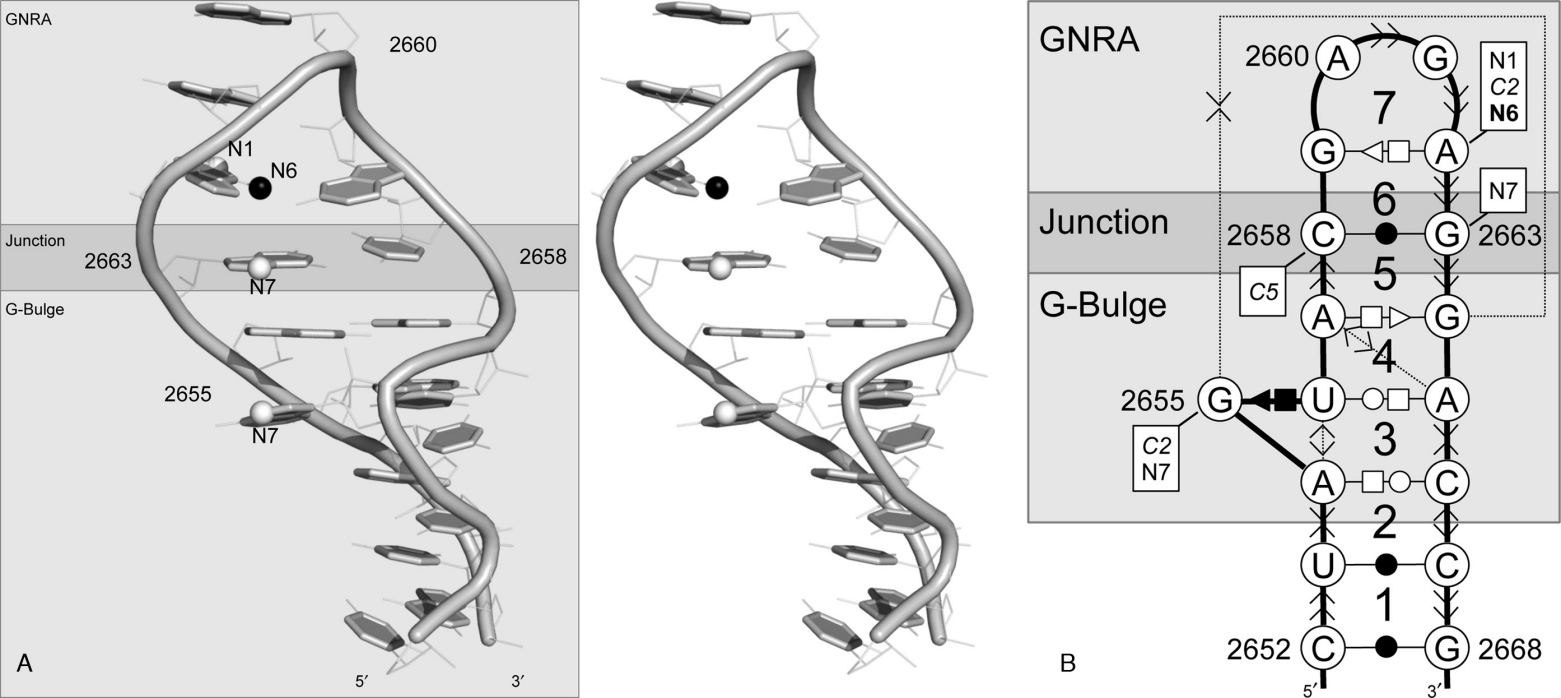
*E. coli* 23S rRNA SRL. (**A**) Stereo view of the SRL 3D structure (PDB ID 2AWB). The base and phosphodiester linkage (cylinder) of each nucleotide are shown in dark gray. Activity determinants identified by MC-3DQSAR are shown with spheres, where the donor groups are shown in black and bold labels and acceptors in light gray and regular labels. Neutral determinants are not shown. (**B**) Secondary structure and NCMs. The NCMs are numbered 1–7. The backbone is shown using bold lines; the base pairing interactions are shown using the Leontis and Westhof nomenclature; and the base stacking interactions are shown using the Major and Thibault nomenclature. The activity determinants identified by MC-3DQSAR are shown in boxes. The identity of the atomic groups from the seed sequence is shown in bold for donor, italic for neutral and regular for acceptor determinants.

The SRL 3D structure of the *E. coli* 23S rRNA (PDB ID 2AWB, nucleotides B2652–B2668) is often used as the reference of bacterial SRLs. SRL structures maintain a series of base pairing and stacking interactions that were previously reported (37). The crystal structure of the *E. coli* SRL includes seven nucleotide cyclic motifs (NCMs) (13,38), which create three distinctive components (Figure 2). NCM 7 is the well-studied GNRA tetraloop (38–41), and the NCMs 2 to 4 form the composite G-bulge motif. The GNRA and G-bulge motifs are connected by NCM #5, which includes a Watson–Crick base pair that joins the GNRA and G-bulge (6). The GNRA motif is defined by the N2660-R2661-A2662 base stacking, the flanking *trans* G:A S/H base pair, and the hydrogen bonds between the amino proton of G2659 and phosphate group of A2662, and the 2′OH of G2659 and N7 of G2661 (42). The α-sarcin catalyzes the hydrolysis of the phosphodiester linkage between the R and A nucleotides of the SRL GNRA tetraloop (43), while the ricin favors the depurination of the N (44). The G-bulge includes a typical base triple G2655:U2656:A2665 (*cis* G:U S/H and *trans* U:A W/H).

**Figure 3.**
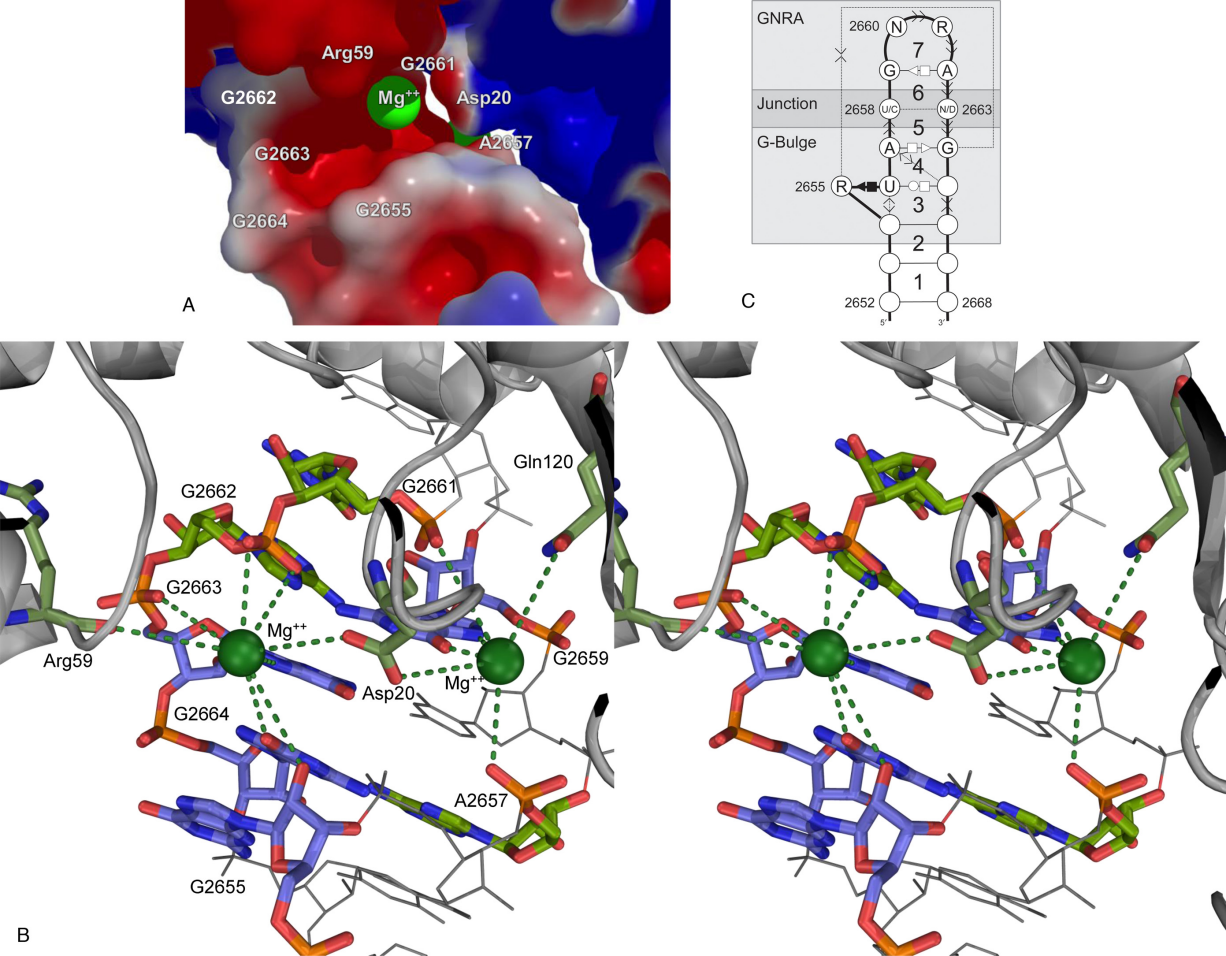
Structural determinants of viable bacterial SRLs. (**A**) Electrostatic surface of the sarcin–ricin/EF-G interaction. Surface potentials are presented in kT/e. The red color indicates negatively charged areas (EF-G from −1; SRL from −8) and blue positively charged areas (EF-G to 1; SRL to 0). The SRL/EF-G recognition put in close contact two negatively charged areas, made possible by the presence of two Mg2+ ions (green spheres). (**B**) Plausible Mg^2+^ coordination between the SRL and EF-G from crystal structures (PDB IDs 4KIX and 4KIY). The green dashed lines indicate possible direct coordination. One of the Mg^2+^ ions (green sphere on the right) is located near atoms Gln120:OE1 and Asp20:OD2 of the EF-G, and A2657:O2P, G2659:O6, G2659:N7 and G2661:O1P of the SRL. The second Mg^2+^ ion (green sphere on the left) is near atoms Arg59:O and Asp20:OD1 of the EF-G, and G2655:O2′, G2662:O1P, G2662:O2P, G2663:N7 and G2664:O6 of the SRL. The EF-G residues and SRL nucleotides involved in Mg^2+^ coordination are shown with colored sticks; others in gray. The bases G2655, G2659, G2663 and G2664 stack and are shown with blue carbons. (**C**) The SRL structural determinants represented as sequence and base interaction constraints. The SRL is composed of the GNRA and G-bulge motifs linked by the Y2658:N2663, but C2658:C2663, junction base pair.

Using MC-3DQSAR, we identified the activity profile of the SRL. Figure 2 shows the determinants identified in the viable profile. As expected, most determinants are located in the GNRA flanking base pair (G:A S/H *trans*), the 2658:2663 (C:G W/W *cis*) junction base pair and the G-bulge (shown as spheres in Figure 2A and in boxes in Figure 2B). In particular, the visualization of a recent crystal structure of the *E. coli* 23S rRNA bound to an EF-GA (45) suggests possible Mg^2+^ coordination involving the O6 and N7 groups of G2659, as well as the N7 group of G2663 (Figure 3). Note that the groups on G2659 are absent in the MC-3DQSAR profile because G is the only nucleotide at this position in the learning set.

In the *E. coli* structure, C2658:G2663 forms a *cis* W/W base pair, which is conserved in all bacterial sequences. Mutagenesis studies determined that the C:G and U:A base pairs at this position confer bacterial viability, whereas G:C and A:U are lethal (7). Corroborating with the mutagenesis studies, MC-3DQSAR identified the missing determinants in the lethal variants (#01 and #03) (Table 1). Based on crystallographic data, Correll and co-workers observed that the SRL fold of the lethal variants is similar to that of the *E. coli* and that an important difference resides in the positions of the 2658:C5 and 2663:N7 groups (6). In our analysis, both of these atomic groups are determinants.

To generalize the rules at the base pair of the junction, we experimentally tested three additional variants: the lethal variant #26 (G:G) and the viable variants #28 (U:C) and #30 (U:U). In the case of variant #26, the model obtained by base substitutions contains serious atomic clashes due to the limited space to accommodate the G:G W/W *cis* base pair. Such clashes occur in the case of any purine:purine base pair at the junction (see Table 1). Removing the clashes necessitates changing the W/W *cis* geometry, and thus the reorganization of the chemical groups, which in this case would not match the activity profile of the SRL. The experimental validation of variant #26 confirmed that no alternative geometry is possible. Interestingly, the viable variants #28 and #30 obtain good scores, respectively, 0.87 and 0.89. The U:U base pair allows for a local reorganization and the substitution of the missing determinant G2663:N7 by U2663:O4, whereas it could be substituted by G2664:O6 in variant #28 (see Figure 3B). These data indicate that three of the four pyrimidine:pyrimidine base pairs at the junction allow for an active SRL in the context of a bacterial ribosome. The C:C base pair is excluded by the lethality of variant #4, and thus a Y:N base pair at the junction, but C:C, is viable. Interestingly, sequence variant #4 is among the few that do not favor the 2D structure of the SRL (Table 1). In Figure 3C, we summarized the 2D structure determinants of a viable bacterial SRL.

The recent crystal structure of the *E. coli* ribosome highlighted two Mg^2+^ ions at the interface with an EF-G (45). The electrostatic surface of this region shows that the cations allow for the interaction between two electronegative regions, respectively, in the SRL and EF-G (Figure 3A). Seven nucleotides of the SRL could possibly participate to the interface between the RNA and EF-G in coordination with the cations, 2655, 2657, 2659 and 2661–2664, and three amino acids of the EF-G, Arg59, Asp20 and Gln120 (Figure 3B).

All variations of nucleotide 2662 (variants #08, #09 and #10) modify the geometry of the GNRA tetraloop by altering the flanking *trans* G:A S/H base pair. The particular geometry of this base pair exposes at the surface the N7 and O6 groups of nucleotide G2659. Removing these groups or changing the G:A geometry would affect the coordination with a cation. Besides, modifying otherwise the tetraloop geometry would alter the position of 2662:O1P, also involved in a possible coordination with the cation. The lethal variants #05 and #07 have pyrimidines at position 2655. This weakens the stacking with nucleotide 2664 and affects the position and orientation of the sugar pucker of 2655 that exposes its 2′OH group and makes a coordination with one of the cations. Stacking is not accounted by MC-3DQSAR, but the determinants N7 and C2 confirms the purine requirement.

### Leadzyme

**Figure 4.**
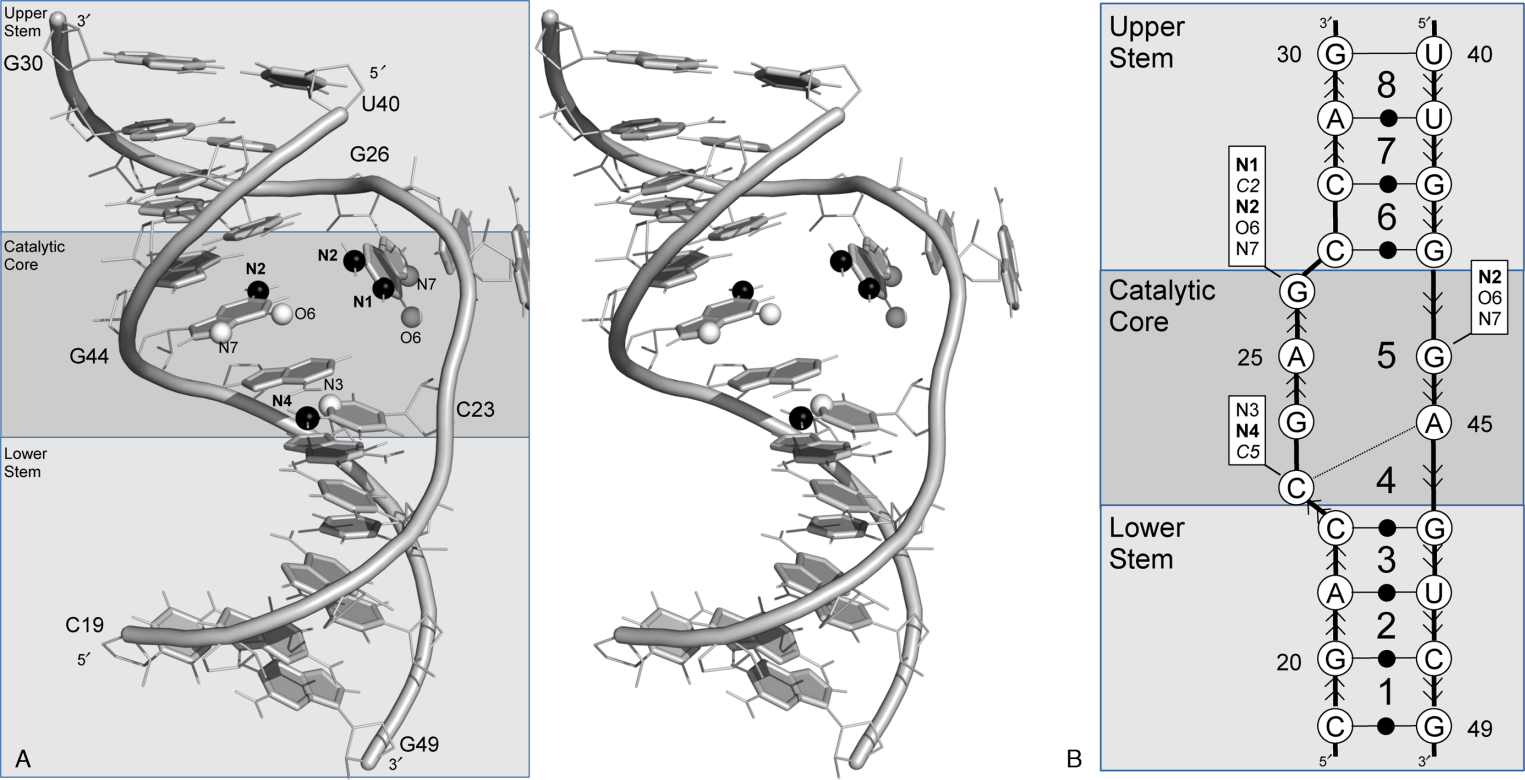
Leadzyme structure. (**A**) Stereo view of the leadzyme 3D structure (PDB ID 1NUJ). Graphical details as in Figure 2A. (**B**) Secondary structure and NCMs (numbered 1–8). Base pairing and stacking nomenclature, and activity determinants are shown as in Figure 2A.

The leadzyme is an auto catalytic RNA that was identified by an *in vitro* selection method of tRNA^Phe^ variants (46). The leadzyme undergoes self-cleavage in the presence of Pb^2+^ and Mg^2+^. The 2D structure is composed of two helical regions (upper and lower stems) surrounding an asymmetric interior loop of four unpaired nucleotides on one strand and two on the other, which constitute the catalytic core. A crystal structure shows a non-canonical C23:A45 base pair in the interior loop (25), and thus the leadzyme is formed by eight NCMs (Figure 4). Two NCMs, #4 and #5, constitute the catalytic core. Interestingly, a proposed pre-catalytic structure based on chemical modification data predicts the formation of a base triple composed of a C23:G44 W/W *cis* and a non-canonical G26:G44 base pair. This suggests the dynamics of this interior loop induces the formation of a triple-based additional NCM prior to catalysis (47,48).

The leadzyme catalytic reaction is carried out in a two-step SN2 mechanism. First, a unique phosphodiester bond is cleaved and results in the formation of a 2′, 3′-cyclic phosphate. Second the hydrolysis of the cyclic phosphate transforms it into a 3′-phosphate. This cleavage reaction occurs between nucleotides 23 and 24 of the catalytic core (Figure 4B). The sequence-activity relationships of the leadzyme have been studied by systematic substitution of chemically modified nucleotides, where essential chemical groups have been identified (49). These experimental data constitute an excellent test to validate MC-3DQSAR. The learning set was made of the seed and 34 sequence variants obtained from a randomized nucleotide substitution study (24). We considered the seed and eight active variants (relative Pb^2+^ cleavage extent ≥ 0.08) and 26 inactive variants (Table 2). We generated a 3D model for each leadzyme variant using base substitution. The 3D models constituted the training set.

Using the training set and MC-3DQSAR, we identified eleven determinants of the leadzyme cleavage activity, which are shown in Figure 4: on nucleotide C23, C5, the acceptor N3 and the donor N4; on G26, C2, the donors N1 and N2 and the acceptors O6 and N7; and on G44, the donor N2, and the acceptors O6 and N7. We compared these determinants to those identified in the catalytic core by systematic chemical modifications (49). The C23:C5 and G26:C2 groups were not tested experimentally. The specific role of these two neutral groups in the structures of active variants is yet to be determined. Nine of the 11 groups on four of the six nucleotides of the catalytic core were identified by both methods. The groups that were only identified experimentally are G24:N7, and A25:N6 and A25:N7. While the experimental data revealed the G24:N7 group crucial for cleavage, the learning set contains active variants with U24 that does not expose the N7 group. This suggests that the role in cleavage of G24:N7 could be substituted by an O4 group, which, however, was outside our detection threshold. While the experimental data revealed that A25:N6 and A25:N7 were involved in the cleavage of the leadzyme, this information could not be deduced from the variants used in the learning set. The A25:N7 is present in all variants and thus is impossible to infer as a determinant by lack of a counter example. The N6 group is absent in the active sequence variant #1, where it is substituted by the G25:O6 group. The acceptor O6 has the opposite role and was thus eliminated from the set of determinants.

### Hammerhead ribozyme

**Figure 5.**
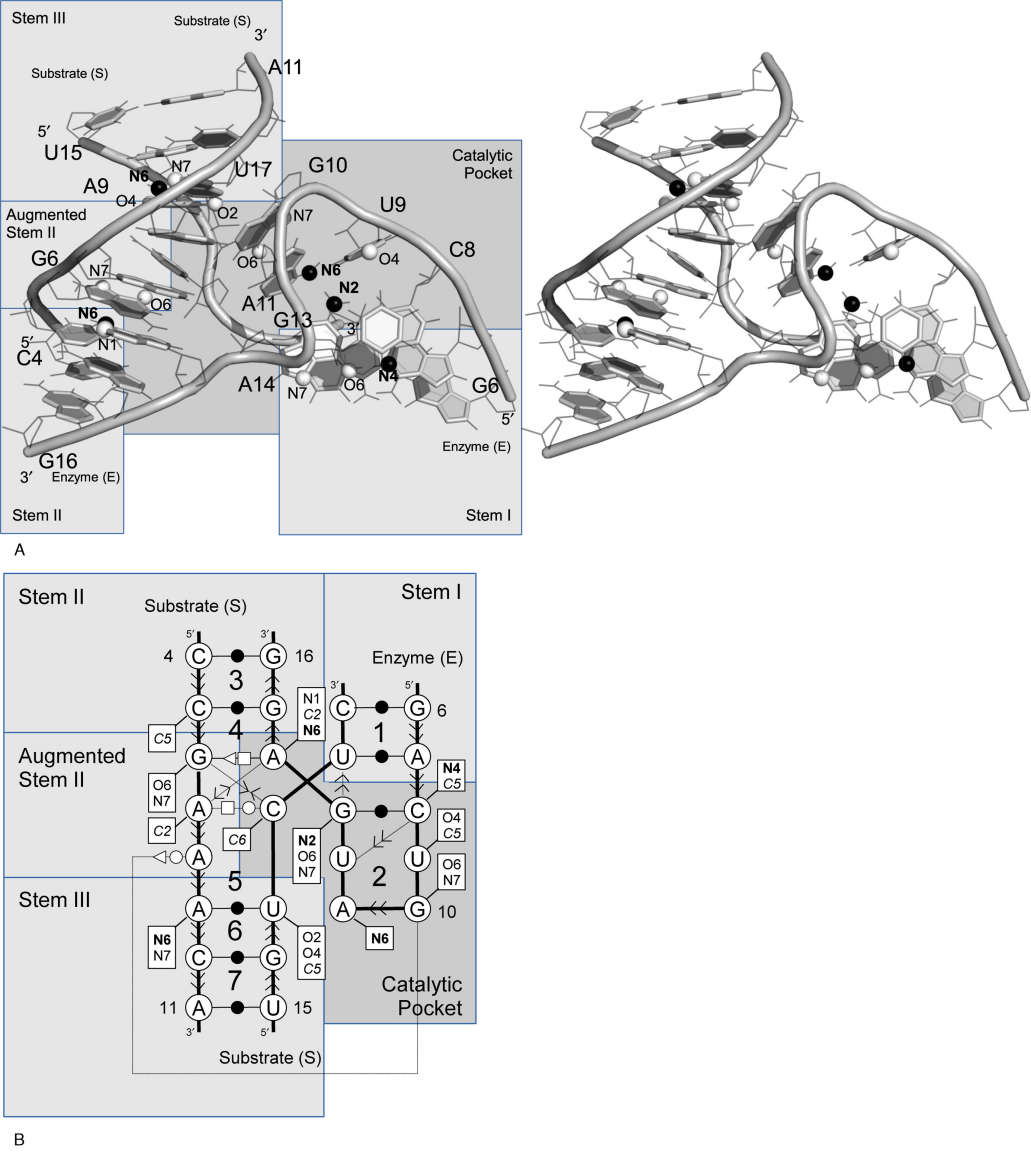
Hammerhead ribozyme structure. (**A**) Stereo view of the hammerhead ribozyme 3D structure (PDB ID 2OEU). Graphical details as in Figure 2A. (**B**) Secondary structure and NCMs (numbered 1–7). Base pairing and stacking nomenclature, and activity determinants are shown as in Figure 2A.

The hammerhead ribozyme undergoes a site-specific cleavage in the presence of Mg^2+^. Its 2D structure is composed of three helices that are joined by a three-way junction catalytic core (50) (Figure 5). The analysis of a hammerhead ribozyme crystal structure reveals it is made of seven NCMs, including three made of the interaction between the enzyme and substrate strands, #1, #3 and #4 (27). A 3D interaction between terminal loops of stems I and II greatly improves the catalytic activity (51,52). The cleavage site is located on the phosphate group between nucleotides 18 and 19 of the substrate strand.

The hammerhead ribozyme has been heavily studied and several mutagenesis and chemical modification studies have identified essential catalytic chemical groups (53). The hammerhead ribozyme represents another excellent example to validate MC-3DQSAR. The learning set was made of the seed sequence and 54 variants taken from a systematic mutagenesis study of the catalytic core (26). We generated a 3D model for each hammerhead ribozyme variant using base substitution. These 3D models constituted the training set.

Using the training set and MC-3DQSAR, we identified 23 determinants of the catalytic activity of the hammerhead ribozyme, which are shown in Figure 5: seven neutral chemical groups; 11 acceptor groups and five donor groups. Ten of the 23 determinants identified by MC-3DQSAR were shown essential for cleavage (53). S(U17):O2 and E(G13):N2 were shown to have little effect on cleavage (*k*_rel_ ≥ 0.2) (53). The acceptor S(U17)O2 is found among the determinants because it is present in all active variants of the learning set. Similarly, the donor E(G13):N2 is captured because there is no variation at position E(13) in the positive examples. The remaining determinants identified by MC-3DQSAR include three acceptor groups, one donor group and seven neutral groups. These groups were not tested experimentally.

Among the groups that were shown crucial for catalysis, but were unidentified by MC-3DQSAR: S(A7):N1 is not exposed to the solvent; E(G13):N3 and S(A7):N3 could not be identified as a determinant by MC-3DQSAR because the N3 group is undetectable by the base substitution building approach since at this position any canonical nucleotide has a donor group: N3 in purines and O4 in pyrimidines, and E(G15):N7 can be substituted by a U:O4 group (variant #13), but was outside our detection threshold.

## DISCUSSION

Our results confirm our hypothesis that current RNA 3D models are required to properly assess the activity levels of RNA molecules. Using atomic superimposition to conserve the position and orientation of the bases from a reference structure was sufficient to build 3D models that are acceptable to be used in a QSAR approach. This was enough 3D structural information, which, in combination with limited mutagenesis data, allowed us to identify a series of structural determinants required to build an interface between an RNA and an implicit moiety. Knowing the 3D structure of at least one positive variant of an RNA family then allows for inferring the sequence interplay conferring activity, which is crucial in designing agents that can target selected variants. For instance, antibiotics targeting undesired bacteria without affecting their hosts.

Many deleterious mutation sites in the ribosome and other non-coding RNAs have been identified, and the need for new antibiotics has prompted the seeking for even more. However, the functional determinants of these sites are generally poorly understood. Their involvement in ribosome structure, folding and assembly, as well as in protein translation mechanisms is difficult to characterize experimentally. The discovery of a deleterious mutation site points to a structural element of the RNA, which after all is a single piece of a complex puzzle. One of the advantages of QSAR approaches is that they focus on a single piece while abstracting the others. For instance, the results we obtained from SRL data alone provided insightful information on structural determinants involved in translation, here the EF-G recognition and binding. The results we obtained for the leadzyme and the hammerhead ribozyme identified structural determinants that were previously revealed by chemical modification studies. The systematic application of MC-3DQSAR to deleterious mutation sites may distinguish those likely to be specific drug targets, i.e. for which we can design potent drugs with sufficient specificity to avoid affecting the viability of the hosts.

## AVAILABILITY

The 3D models used in this study are available online at major.iric.ca/MC-3DQSAR. A web service is provided at major.iric.ca/mc3dqsar, but is currently limited to comparing the profiles of new SRL, leadzyme and hammerhead ribozyme sequence variants.

## References

[1] Phillips L. (2013). Infectious disease: TB's revenge. Nature.

[2] Wilson D.N., Doudna Cate J.H. (2012). The structure and function of the eukaryotic ribosome. Cold Spring Harb. Perspect. Biol..

[3] Dunkle J.A., Cate J.H. (2010). Ribosome structure and dynamics during translocation and termination. Annu. Rev. Biophys..

[4] McCoy L.S., Xie Y., Tor Y. (2011). Antibiotics that target protein synthesis. Wiley interdisciplinary reviews. RNA.

[5] Poehlsgaard J., Douthwaite S. (2005). The bacterial ribosome as a target for antibiotics. Nature reviews. Microbiology.

[6] Correll C.C., Beneken J., Plantinga M.J., Lubbers M., Chan Y.L. (2003). The common and the distinctive features of the bulged-G motif based on a 1.04 A resolution RNA structure. Nucleic Acids Res..

[7] Chan Y.L., Sitikov A.S., Wool I.G. (2000). The phenotype of mutations of the base-pair C2658.G2663 that closes the tetraloop in the sarcin/ricin domain of Escherichia coli 23 S ribosomal RNA. J. Mol. Biol..

[8] Moazed D., Robertson J.M., Noller H.F. (1988). Interaction of elongation factors EF-G and EF-Tu with a conserved loop in 23S RNA. Nature.

[9] Das R., Karanicolas J., Baker D. (2010). Atomic accuracy in predicting and designing noncanonical RNA structure. Nat. Methods.

[10] Ding F., Sharma S., Chalasani P., Demidov V.V., Broude N.E., Dokholyan N.V. (2008). Ab initio RNA folding by discrete molecular dynamics: from structure prediction to folding mechanisms. RNA.

[11] Jonikas M.A., Radmer R.J., Laederach A., Das R., Pearlman S., Herschlag D., Altman R.B. (2009). Coarse-grained modeling of large RNA molecules with knowledge-based potentials and structural filters. RNA.

[12] Cao S., Chen S.J. (2011). Physics-based de novo prediction of RNA 3D structures. J. Phys. Chem..

[13] Parisien M., Major F. (2008). The MC-Fold and MC-Sym pipeline infers RNA structure from sequence data. Nature.

[14] Rother M., Rother K., Puton T., Bujnicki J.M. (2011). ModeRNA: a tool for comparative modeling of RNA 3D structure. Nucleic Acids Res.

[15] Caballero J., Fernandez L., Abreu J.I., Fernandez M. (2006). Amino Acid Sequence Autocorrelation vectors and ensembles of Bayesian-Regularized Genetic Neural Networks for prediction of conformational stability of human lysozyme mutants. J. Chem. Inf. Model..

[16] Cabrera M.A., Gonzalez I., Fernandez C., Navarro C., Bermejo M. (2006). A topological substructural approach for the prediction of P-glycoprotein substrates. J. Pharm. Sci..

[17] Perez Gonzalez M., Gonzalez Diaz H., Molina Ruiz R., Cabrera M.A., Ramos de Armas R. (2003). TOPS-MODE based QSARs derived from heterogeneous series of compounds. Applications to the design of new herbicides. J. Chem. Inf. Comput. Sci..

[18] Xiao Z., Xiao Y.D., Feng J., Golbraikh A., Tropsha A., Lee K.H. (2002). Antitumor agents. 213. Modeling of epipodophyllotoxin derivatives using variable selection k nearest neighbor QSAR method. J. Med. Chem..

[19] Helguera A.M., Rodriguez-Borges J.E., Garcia-Mera X., Fernandez F., Cordeiro M.N. (2007). Probing the anticancer activity of nucleoside analogues: a QSAR model approach using an internally consistent training set. J. Med. Chem..

[20] Marrero-Ponce Y., Marrero R.M., Torrens F., Martinez Y., Bernal M.G., Zaldivar V.R., Castro E.A., Abalo R.G. (2006). Non-stochastic and stochastic linear indices of the molecular pseudograph's atom-adjacency matrix: a novel approach for computational in silico screening and ‘rational’ selection of new lead antibacterial agents. J. Mol. Model..

[21] Gonzalez-Diaz H., Vilar S., Santana L., Podda G., Uriarte E. (2007). On the applicability of QSAR for recognition of miRNA bioorganic structures at early stages of organism and cell development: embryo and stem cells. Bioorg. Med. Chem..

[22] Cannone J.J., Subramanian S., Schnare M.N., Collett J.R., D'Souza L.M., Du Y., Feng B., Lin N., Madabusi L.V., Muller K.M. (2002). The comparative RNA web (CRW) site: an online database of comparative sequence and structure information for ribosomal, intron, and other RNAs. BMC Bioinformatics.

[23] Szewczak A.A., Moore P.B. (1995). The sarcin/ricin loop, a modular RNA.. J. Mol. Biol..

[24] Pan T., Dichtl B., Uhlenbeck O.C. (1994). Properties of an in vitro selected Pb2+ cleavage motif. Biochemistry.

[25] Wedekind J.E., McKay D.B. (2003). Crystal structure of the leadzyme at 1.8 A resolution: metal ion binding and the implications for catalytic mechanism and allo site ion regulation. Biochemistry.

[26] Ruffner D.E., Stormo G.D., Uhlenbeck O.C. (1990). Sequence requirements of the hammerhead RNA self-cleavage reaction. Biochemistry.

[27] Martick M., Lee T.S., York D.M., Scott W.G. (2008). Solvent structure and hammerhead ribozyme catalysis. Chem. Biol..

[28] Chan Y.L., Wool I.G. (2008). The integrity of the sarcin/ricin domain of 23 S ribosomal RNA is not required for elongation factor-independent peptide synthesis. J. Mol. Biol..

[29] Chan Y.-L., Dresios J., Wool I.G. (2006). A pathway for the transmission of allosteric signals in the ribosome through a network of RNA tertiary interactions. J. Mol. Biol..

[30] Macbeth M.R., Wool I.G. (1999). The phenotype of mutations of G2655 in the sarcin/ricin domain of 23 S ribosomal RNA. J. Mol. Biol..

[31] Leontis N.B., Westhof E. (2001). Geometric nomenclature and classification of RNA base pairs. RNA.

[32] Major F., Thibault P., Lengauer T (2007). Computer Modeling of RNA Three-Dimensional Structures. Bioinformatics: From Genomes to Therapies.

[33] DeLano W.L. (2002). DeLano Scientific. The PyMOL Molecular Graphics System.

[34] Dolinsky T.J., Czodrowski P., Li H., Nielsen J.E., Jensen J.H., Klebe G., Baker N.A. (2007). PDB2PQR: expanding and upgrading automated preparation of biomolecular structures for molecular simulations. Nucleic Acids Res..

[35] Dolinsky T.J., Nielsen J.E., McCammon J.A., Baker N.A. (2004). PDB2PQR: an automated pipeline for the setup of Poisson-Boltzmann electrostatics calculations. Nucleic Acids Res..

[36] Baker N.A., Sept D., Joseph S., Holst M.J., McCammon J.A. (2001). Electrostatics of nanosystems: application to microtubules and the ribosome. Proc. Natl Acad. Sci. U.S.A..

[37] Spackova N.a., Sponer J. (2006). Molecular dynamics simulations of sarcin-ricin rRNA motif. Nucleic Acids Res..

[38] Lemieux S., Major F. (2006). Automated extraction and classification of RNA tertiary structure cyclic motifs. Nucleic Acids Res..

[39] Woese C.R., Winker S., Gutell R.R. (1990). Architecture of ribosomal RNA: constraints on the sequence of ‘tetra-loops’. Proc. Natl Acad. Sci. U.S.A..

[40] Heus H.A., Pardi A. (1991). Structural features that give rise to the unusual stability of RNA hairpins containing GNRA loops. Science.

[41] Jaeger L., Michel F., Westhof E. (1994). Involvement of a GNRA tetraloop in long-range RNA tertiary interactions. J. Mol. Biol..

[42] Jucker F.M., Pardi A. (1995). GNRA tetraloops make a U-turn. RNA.

[43] Endo Y., Wool I.G. (1982). The site of action of alpha-sarcin on eukaryotic ribosomes. The sequence at the alpha-sarcin cleavage site in 28 S ribosomal ribonucleic acid. J. Biol. Chem..

[44] Endo Y., Mitsui K., Motizuki M., Tsurugi K. (1987). The mechanism of action of ricin and related toxic lectins on eukaryotic ribosomes. The site and the characteristics of the modification in 28 S ribosomal RNA caused by the toxins. J. Biol. Chem..

[45] Pulk A., Cate J.H. (2013). Control of ribosomal subunit rotation by elongation factor G. Science.

[46] Pan T., Uhlenbeck O.C. (1992). A small metalloribozyme with a two-step mechanism. Nature.

[47] Lemieux S., Chartrand P., Cedergren R., Major F. (1998). Modeling active RNA structures using the intersection of conformational space: application to the lead-activated ribozyme. RNA.

[48] Yajima R., Proctor D.J., Kierzek R., Kierzek E., Bevilacqua P.C. (2007). A conformationally restricted guanosine analog reveals the catalytic relevance of three structures of an RNA enzyme. Chem. Biol..

[49] Chartrand P., Usman N., Cedergren R. (1997). Effect of structural modifications on the activity of the leadzyme. Biochemistry.

[50] Forster A.C., Symons R.H. (1987). Self-cleavage of plus and minus RNAs of a virusoid and a structural model for the active sites. Cell.

[51] De la Pena M., Gago S., Flores R. (2003). Peripheral regions of natural hammerhead ribozymes greatly increase their self-cleavage activity. EMBO J..

[52] Khvorova A., Lescoute A., Westhof E., Jayasena S.D. (2003). Sequence elements outside the hammerhead ribozyme catalytic core enable intracellular activity. Nat. Struct. Mol. Biol..

[53] Nelson J.A., Uhlenbeck O.C. (2008). Hammerhead redux: does the new structure fit the old biochemical data?. RNA.

